# Supplementary material for: Creatinine-to-Cystatin C Ratio Combined with FIB-4 and ELF for Noninvasive Fibrosis Assessment in MASLD
Source: Int J Mol Sci. 2025 Sep 30;26(19):9560. doi: 10.3390/ijms26199560 (PMC12524340; doi:10.3390/ijms26199560)

Figure S1
Association between CCR and ballooning
CCR values according to hepatocyte ballooning grade (0–2). No significant difference was found (p = 0.15).

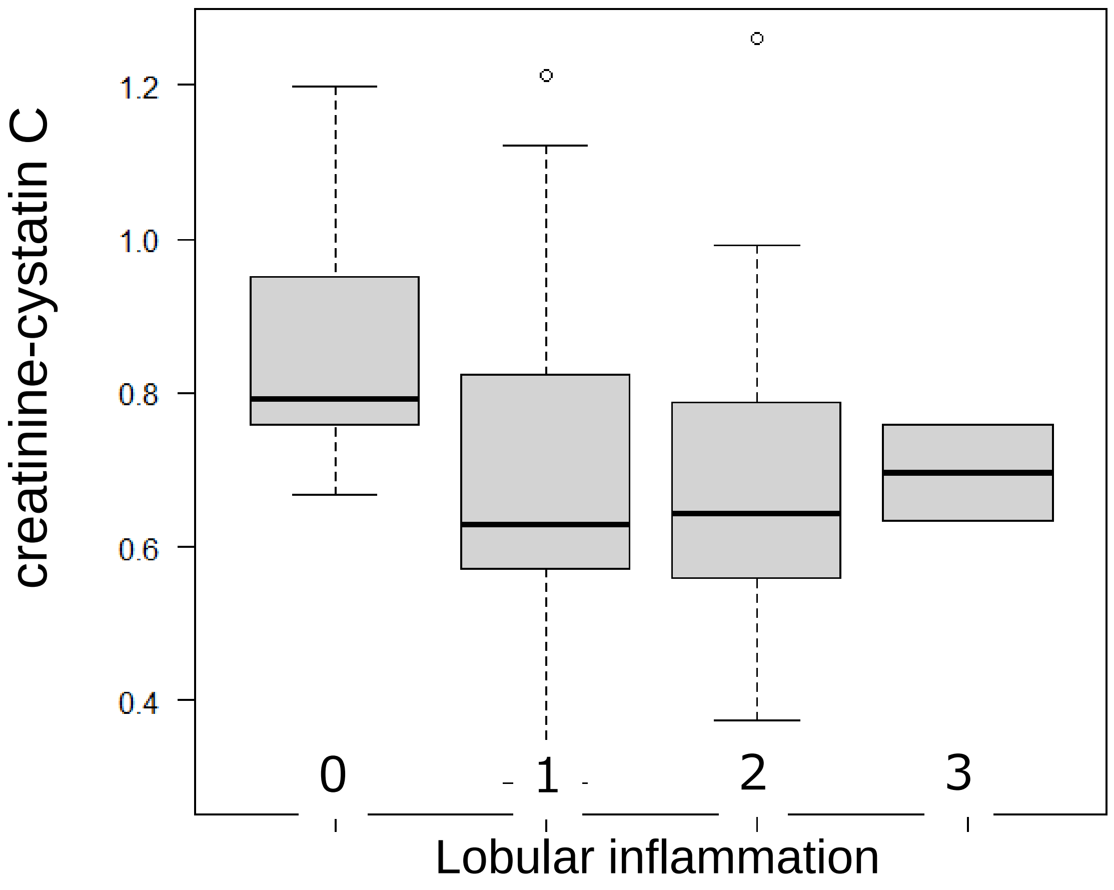

Figure S2
Association between CCR and lobular inflammation
CCR values according to lobular inflammation grade (0–2). The difference approached but did not reach statistical significance (p = 0.0533).

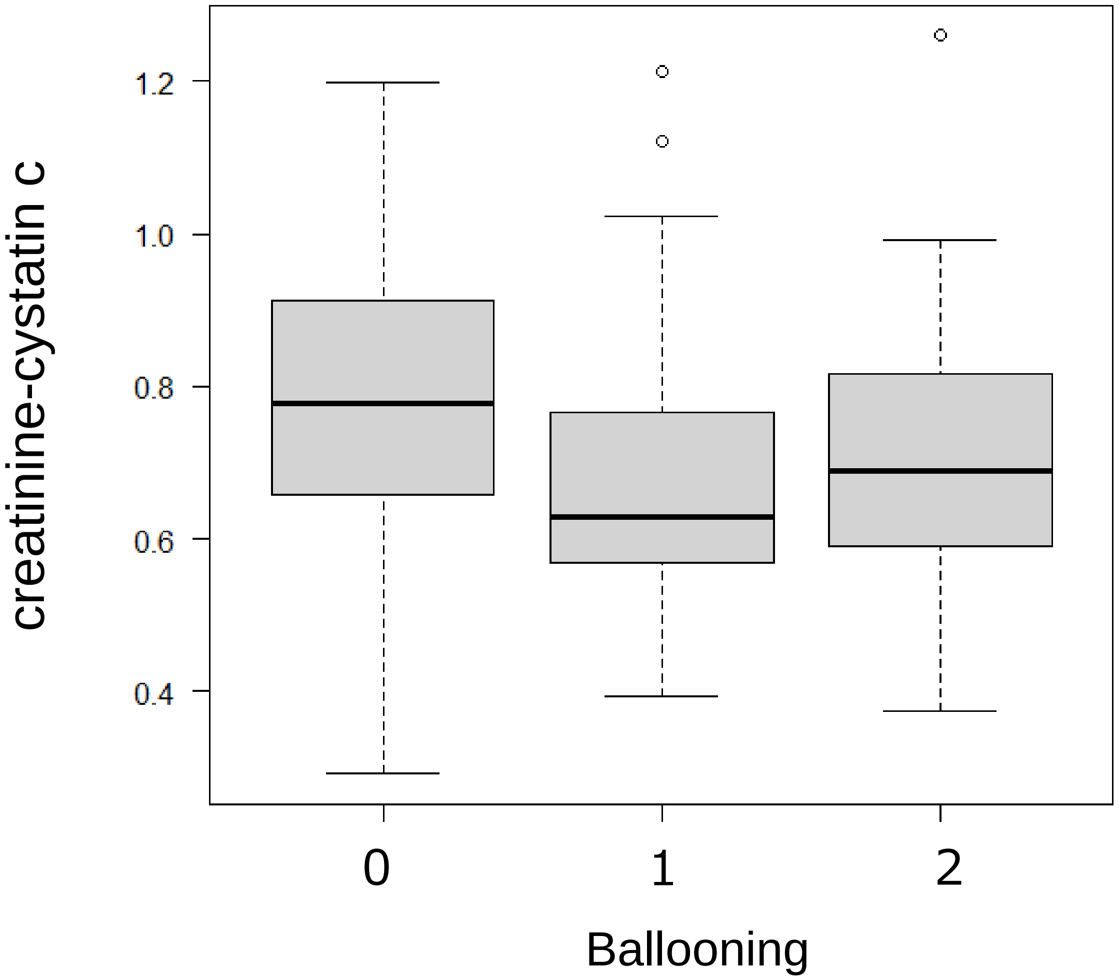

Figure S3

Association between creatinine-to-cystatin C ratio (CCR) and steatosis grade

CCR was compared across steatosis grades (0–3). No statistically significant difference was observed (p = 0.335).

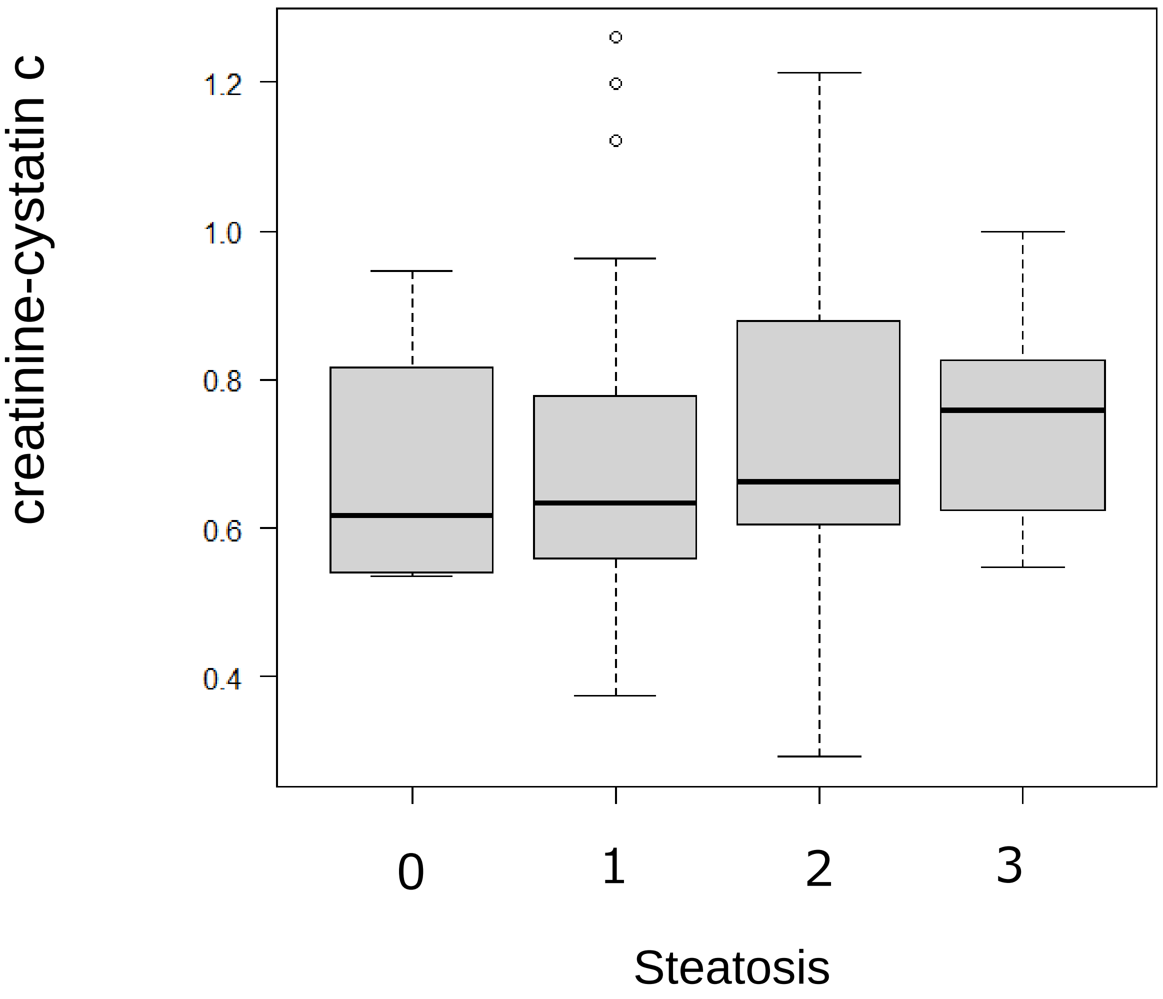

Figure S4
Association between cystatin C and fibrosis stage
Cystatin C levels plotted against fibrosis stage (F0–F4). No statistically significant association was observed (p = 0.289).

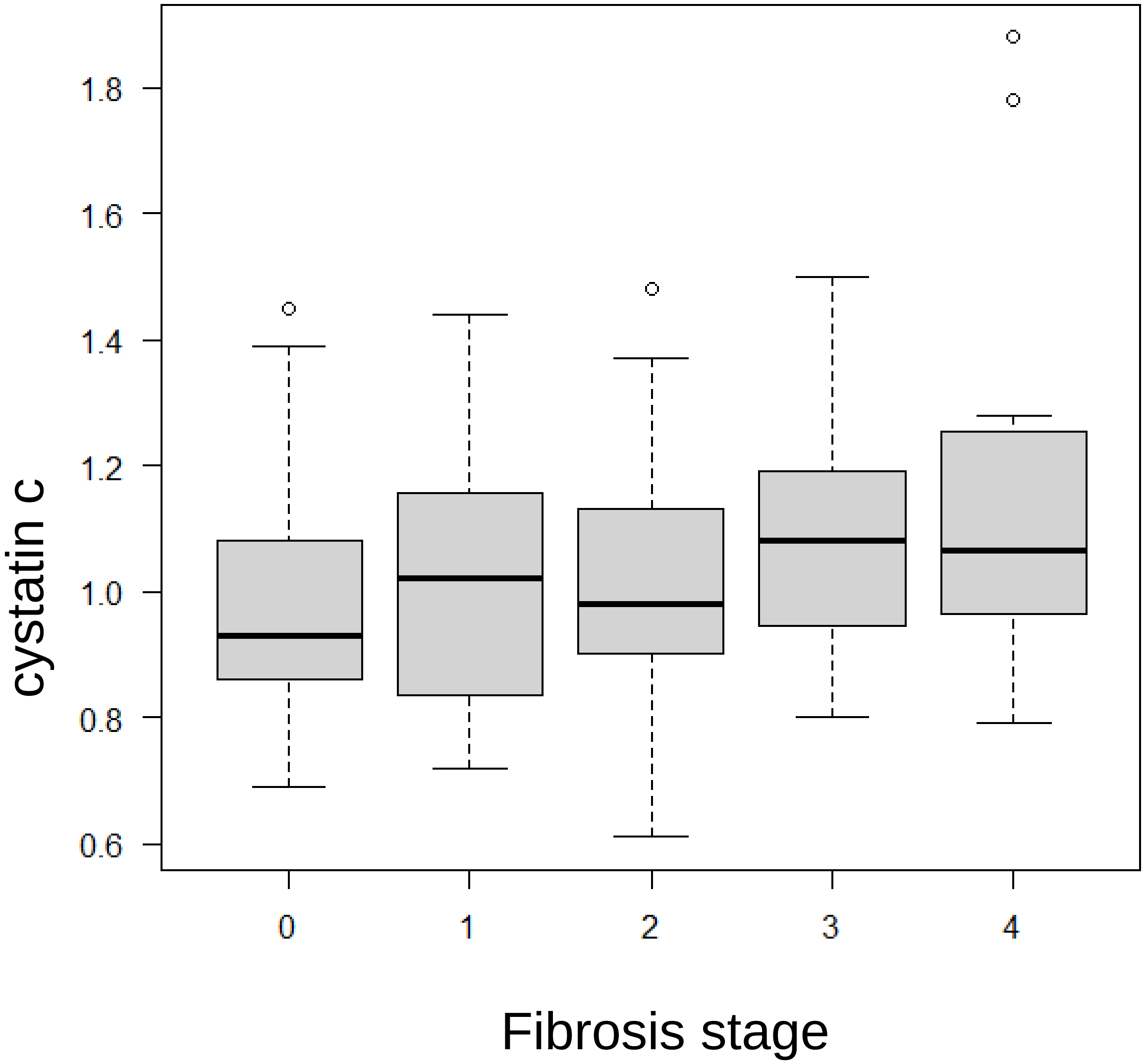

Figure S5
Association between cystatin C and steatosis grade
Cystatin C levels across steatosis grades (0–3). No significant difference was detected ($p = 0.589$).

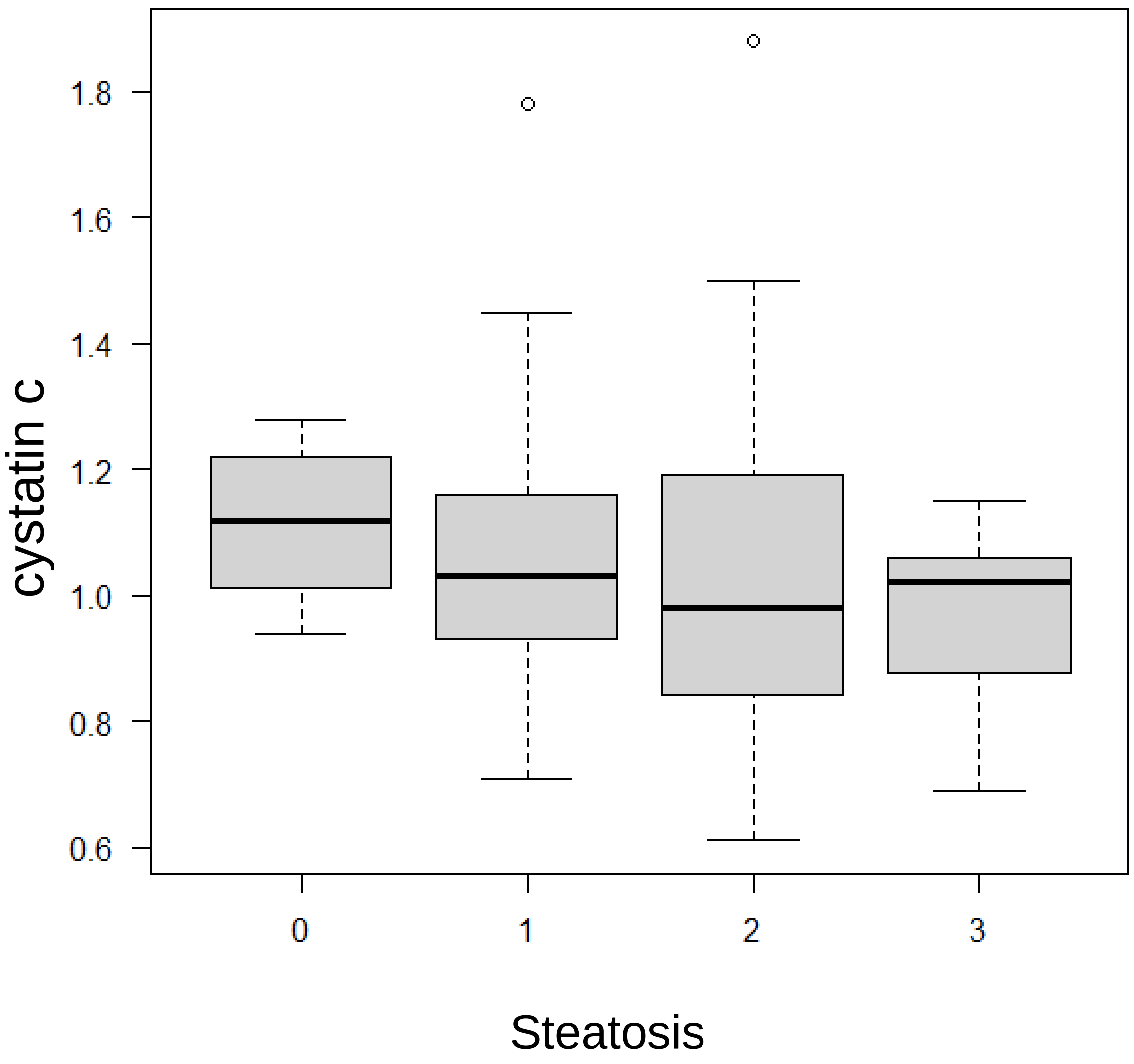

Figure S6

Association between cystatin C and lobular inflammation

Cystatin C values according to lobular inflammation grade (0–2). No significant difference was observed (p = 0.152).

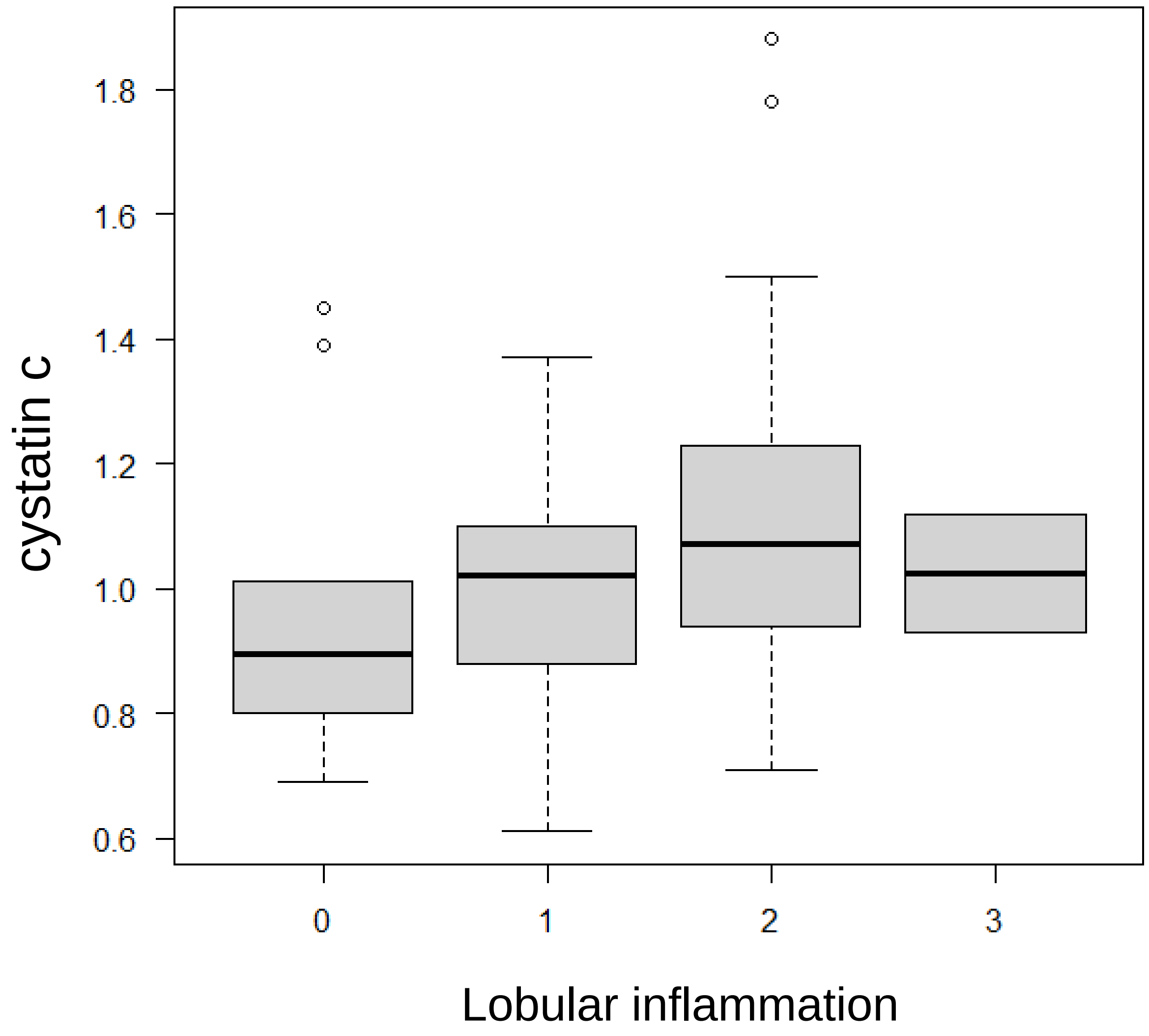

Figure S7

Association between cystatin C and ballooning

Cystatin C levels across hepatocyte ballooning grades (0–2). No significant difference was observed (p = 0.849)

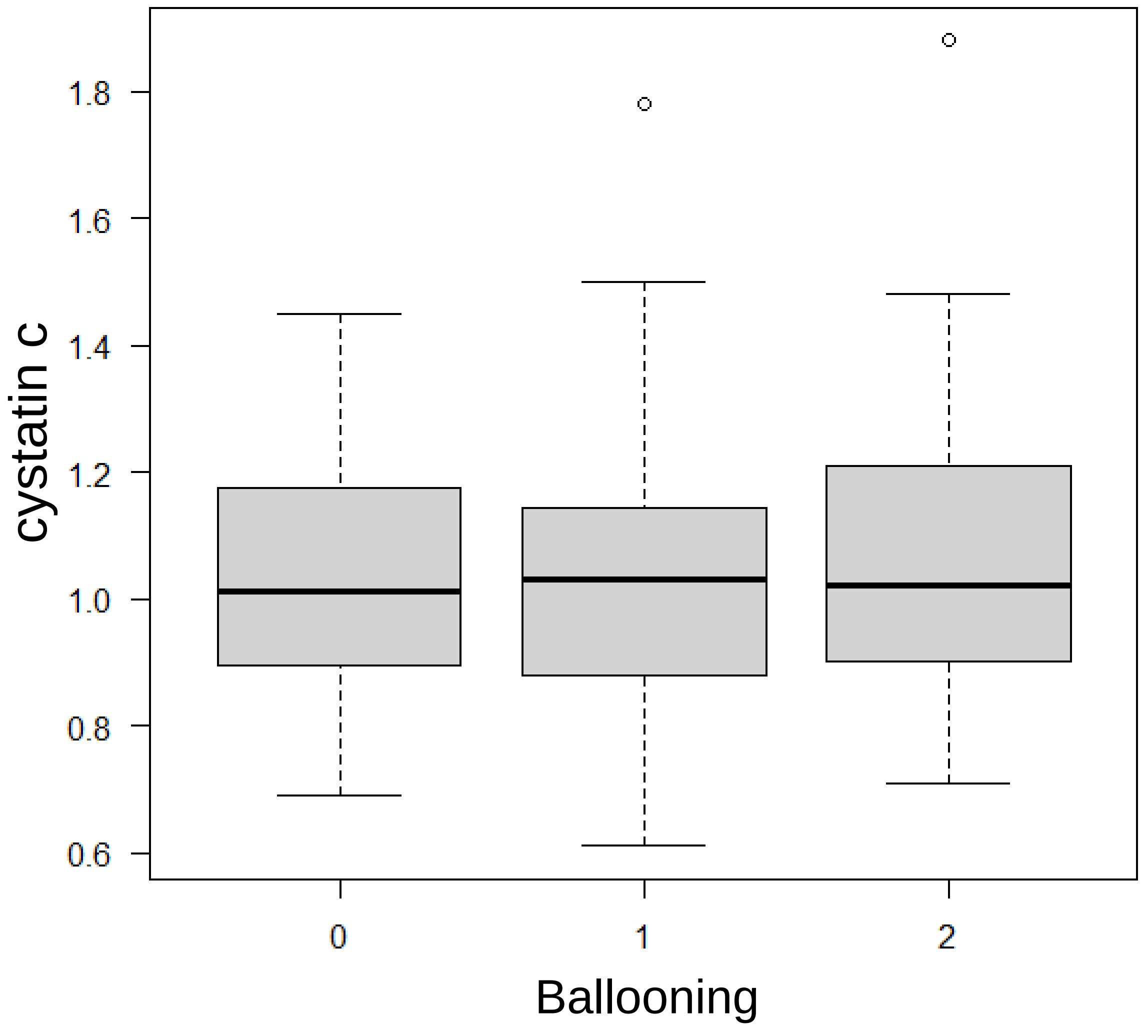

Supplement: Supplementary file 1 [file ijms-26-09560-s001.zip › ijms-3858228-supplementary.pdf]
